# The Cerebral Surfactant System and Its Alteration in Hydrocephalic Conditions

**DOI:** 10.1371/journal.pone.0160680

**Published:** 2016-09-22

**Authors:** Stefan Schob, Donald Lobsien, Benjamin Friedrich, Matthias K. Bernhard, Corinna Gebauer, Julia Dieckow, Matthias Gawlitza, Mandy Pirlich, Dorothee Saur, Lars Bräuer, Ingo Bechmann, Karl-Titus Hoffmann, Cynthia V. Mahr, Ulf Nestler, Matthias Preuß

**Affiliations:** 1 Department of Neuroradiology, University Leipzig, Germany; 2 Department of Neurosurgery, University Leipzig, Germany; 3 Department of Diagnostic and Interventional Radiology, University Leipzig, Germany; 4 Division of Neuropediatrics, University Leipzig, Germany; 5 Department of Neonatology, University Leipzig, Germany; 6 Department of Ophthalmology, University Leipzig, Germany; 7 Department of Neurology, University Leipzig, Germany; 8 Institute of Anatomy, University Leipzig, Germany; 9 Institute of Anatomy, University of Erlangen-Nuremberg, Germany; Universitatsklinikum Freiburg, GERMANY

## Abstract

**Introduction:**

Pulmonary Surfactant reduces surface tension in the terminal airways thus facilitating breathing and contributes to host’s innate immunity. Surfactant Proteins (SP) A, B, C and D were recently identified as inherent proteins of the CNS. Aim of the study was to investigate cerebrospinal fluid (CSF) SP levels in hydrocephalus patients compared to normal subjects.

**Patients and Methods:**

CSF SP A-D levels were quantified using commercially available ELISA kits in 126 patients (0–84 years, mean 39 years). 60 patients without CNS pathologies served as a control group. Hydrocephalus patients were separated in aqueductal stenosis (AQS, n = 24), acute hydrocephalus without aqueductal stenosis (acute HC w/o AQS, n = 16) and idiopathic normal pressure hydrocephalus (NPH, n = 20). Furthermore, six patients with pseudotumor cerebri were investigated.

**Results:**

SP A—D are present under physiological conditions in human CSF. SP-A is elevated in diseases accompanied by ventricular enlargement (AQS, acute HC w/o AQS) in a significant manner (0.67, 1.21 vs 0.38 ng/ml in control, p<0.001). SP-C is also elevated in hydrocephalic conditions (AQS, acute HC w/o AQS; 0.87, 1.71 vs. 0.48 ng/ml in controls, p<0.001) and in Pseudotumor cerebri (1.26 vs. 0.48 ng/ml in controls, p<0.01). SP-B and SP-D did not show significant alterations.

**Conclusion:**

The present study confirms the presence of SPs in human CSF. There are significant changes of SP-A and SP-C levels in diseases affecting brain water circulation and elevation of intracranial pressure. Cause of the alterations, underlying regulatory mechanisms, as well as diagnostic and therapeutic consequences of cerebral SP’s requires further thorough investigations.

## Introduction

Surfactant proteins (SPs) are part of the pulmonary surfactant, a thin layer covering the alveolar surface serving three main purposes (i) decreasing the surface tension at the air-tissue interface to prevent the collapse of the small airways at the end of expiration (ii) facilitating the clearance of airborne pathogens and (iii) regulating the local innate and adaptive immune response [1,2]. The pulmonary surfactant consists of approximately 90% lipids and 10% surfactant proteins A, B, C and D [3]. Both components–lipids and proteins are essential for surfactant functionality [1,2,4]. SPs can be subdivided into two different groups regarding both—structure and mode of action: the relatively large hydrophilic collectines (SP-A and SP-D) and the much smaller, highly hydrophobic proteins SP-B and SP-C.

Surfactant protein A (SP-A) and surfactant protein D (SP-D) help to maintain the physicochemical properties of the surfactant layer. Furthermore, both molecules are opsonins, facilitating the elimination of invading pathogens and dead cells in the lungs and other organs [3,5]. SP-A and SP-D therefore have been considered as pre-assembled, broad-spectrum antibodies of the innate immune system [5].

The hydrophobic proteins SP-B and SP-C strongly interact with phospholipids, thus forming and stabilizing the pulmonary surfactant layer [6–8]. Lack of SP-B and / or SP-C leads to an increase of intraalveolar surface tension, resulting in endexspiratory collapse of the distal airways, atelectasis and finally respiratory distress syndrome, which can be treated with surfactant preparations [9]. In fact the use of surfactant protein containing preparations reduced mortality of respiratory distress syndrome of neonates by approximately 50% [9]

Recently our group detected surfactant proteins as inherent proteins of the CNS [10]. The distribution patterns of SP-A and SP-D were slightly different from SP-B and SP-C. The more rheologically active SP-B and SP-C were detected in choroid plexus and ependymal cells of the brain and spinal canal, representing the major sites of CSF formation and the CSF–tissue interface. The opsonins SP-A and SP-D were found at the sites of the blood-brain and the blood-CSF barrier, respectively [10]. Furthermore the SPs were also found in significant concentrations in the CSF.

CSF net flow comprises of a pulsatile convective flow of different frequencies (e.g. heart cycle and breathing), diffusion and active transport across barriers of the CNS [11]. Since SP modulate the rheological properties of the fluid layer within the distal airways, they might contribute to the regulation of CSF flow. As a consequence, different hydrocephalic conditions might show an altered cerebral SP homeostasis.

Therefore, the aim of the present study was to analyze differences in CSF-SP levels between normal subjects and patients suffering from idiopathic normal pressure hydrocephalus (NPH), aqueductal stenosis (AQS), acute hydrocephalus without aqueductal stenosis (acute HC w/o AQS) and pseudotumor cerebri (PC).

## Patients and Methods

### Patients

CSF specimens of 126 patients were examined. All patients or caregivers gave their written informed consent for the scientific use of CSF-samples and analysis of clinical and radiological data. The study was approved by the local ethics committee (Ethikkommission Universität Leipzig Az 330-13-18112013). Specimens of 60 subjects without conclusive proof of neurological pathologies specimens were used as control group. Those specimens were obtained during the diagnostic workup that necessitated CSF examination by lumbar puncture (e.g. exclusion of subarachnoid hemorrhage, demyelinating disease, meningitis). Furthermore CSF samples were obtained from 66 patients that underwent diagnostic workup and treatment of hydrocephalus or pseudotumor cerebri. Hydrocephalus patients were categorized into the following pathophysiological entities after the review of patient records and brain imaging: aqueductal stenosis (AQS), acute hydrocephalus without aqueductal stenosis (acute HC w/o AQS) and normal pressure hydrocephalus.

24 patients who revealed narrowing or obstruction of the Sylvian aqueduct with corresponding enlargement of the lateral ventricles and the 3^rd^ ventricle in cranial imaging were classified as AQS [12].

20 Patients with the classic triad of symptoms of idiopathic NPH (gait disturbance, urinary incontinence, dementia) and typical morphological criteria in brain imaging (ventriculomegaly and tight convexity sulci in combination with enlarged Sylvian fissures) were included after standardized clinical and radiological examination [11,13,14,15,16]. The group of patients with acute hydrocephalus without aqueductal stenosis (Acute HC w/o AQS, n = 16) was a heterogenous group of patients with acute hydrocephalus of different pathophysiology: posthemorrhagic, postinfectious and idiopathic hydrocephalus without radiological proof of occlusion of ventricular CSF outflow (also termed communicating hydrocephalus). All acute HC without AQS patients presented signs of elevated intracranial pressure. Furthermore six patients meeting the criteria for pseudotumor cerebri (PC) were included [17].

### Quantification of Surfactant Proteins in CSF

Quantification of surfactant protein concentrations was performed using enzyme-linked immunosorbent assays (ELISA) according the manufacturers manual. Commercially available enzyme-linked immunosorbent assay kits (USCN, Wuhan, China) were used to quantify the amount of SP-A (E90890Hu, ELISA Kit for Surfactant Associated Protein A), SP-B (E91622Hu, ELISA Kit for Surfactant Associated Protein B), SP-C (E91623Hu, ELISA Kit for Surfactant Associated Protein C) and SP-D (E91039Hu, ELISA Kit for Surfactant-Associated Protein D) in CSF samples. The analysis was performed using a microplate spectrophotometer (ELISA-reader) at a wavelength of 450 nm and a reference wavelenght of 405 nm for measuring the absorbance. Surfactant protein concentration in ng/ml CSF was calculated by comparison between standard series and the determined values of antigen concentration (protein concentration) according to the manufacturers manual. CSF concentrations of SP-A, SP-C and SP-D lay well above the detection limit of the ELISA kits. Detection limits according the manufacturers manual were as follows; SP-A: 18.27 pg/ml (0.0183 ng/ml), SP-C: 0.126 ng/ml and SP-D: 2.55 ng/ml. CSF concentrations of SP-B of most samples were slightly below the detection limit of the ELISA kit according the manufacturers manual (SP-B: 0.62 ng/ml).

### Further CSF analysis

Routine CSF laboratory data (bacterial cultures, cell count, CSF lactate and glucose concentrations, total CSF protein and protein electrophoresis) were obtained to rule out infection or other inflammatory and autoimmune diseases. Patients with CSF infection were excluded.

### Statistical analysis

Statistical analysis was performed using SPSS Version 22. Data was tested for normality using Shapiro-Wilks test. Differences between groups were analyzed using analysis of variance on ranks with a Dunnett post-hoc analysis. Correlation between age and the concentration of SPs was calculated using a Spearman Rho correlation since SP values were not normally distributed. Significance level was set to 0.05.

## Results

CSF samples of 126 patients were analyzed for Surfactant proteins A, B, C and D. Routine CSF examinations as described above were also performed. An overview of patient demographics is given in Table 1.

**Table 1:** Overview of demographic data of the patient subgroups.

	Control	AQS	Acute HC w/o AQS	NPH	Pseudotumor cerebri
n	60	24	16	20	6
age (yrs)	43.5 (0–84)	19.3 (0–65)	14.5 (0–75)	67.2 (31–84)	25.7 (6–48)
Sex (m/f)	34/26	9/15	6/10	12/8	1/5

**Fig 1:**
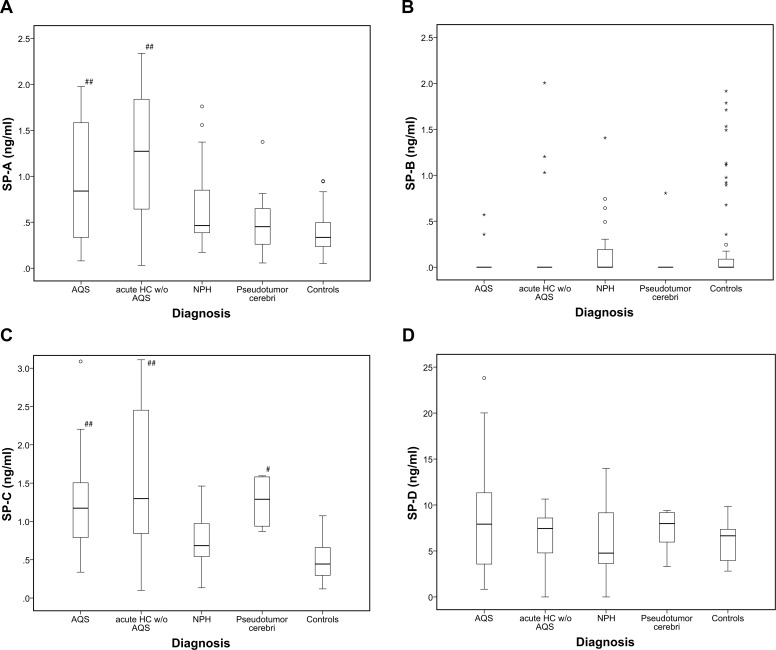
a) CSF levels of SP-A (ng/ml) in control group and hydrocephalus subgroups. In acute hydrocephalic conditions with elevated intracranial pressure (ICP), SP-A levels are significantly elevated. b) CSF levels of SP-B (ng/ml) in control group and hydrocephalus subgroups. SP-B is not significantly altered in hydrocephalus groups compared to control. SP-B concentrations in most specimens were below the detection limit. Detectable SP-B values showed a wide range of variation. c) CSF levels of SP-C (ng/ml) in control group and hydrocephalus subgroups. Compared to controls, SP-C is significantly increased in subgroups with elevated ICP. d) CSF levels of SP-D (ng/ml) in control group and hydrocephalus subgroups. SP-D is present under normal and pathological conditions, there are no significant differences between the subgroups of AQS and controls. Significance levels: # p<0.05 ## p<0.001; all vs. Control. *: Data value lies >3 times of the interquartile range away from the mean value. °: Data value lies between 1.5 and 3x of the interquartile range away from the median value.

Control patients with an age ranging from 14 days to 84 years (mean 43,5 years) were investigated. Under normal conditions, Surfactant proteins A, B, C and D are present in CSF independent from gender. SP-B concentrations in the majority of CSF samples (n = 109) were slightly below the guaranteed detection limit of the ELISA kit. 17 CSF samples revealed SP-B concentrations within the detection range (control group: n = 12; acute HC without AQS: n = 3; NPH: n = 1; pseudotumor cerebri: n = 1). Those specimens with values above the detection range are summarized graphically in Fig 1B. Detectablte SP-B values revealed a wide range of variation, no trend was observable in the investigated subgroups. Statistical comparison between the groups was therefore not applicable. An overview of mean surfactant protein concentrations is shown in Table 2.

**Table 2:** Overview of mean Surfactant Protein A-D levels (ng/ml) and CSF cell count (CC), CSF lactate (lac), CSF glucose (glu) and total CSF protein concentrations (g/l) of various types of CSF disturbances and control group and their respective 95% confidence intervals. * p<0.05, ***p<0.001; all vs. Control

	Control	AQS	Acute HC w/o AQS	NPH	Pseudotumor cerebri
n	60	24	16	20	6
SP-A	0.38 (0.32–0.43)	0.67 (0.57–1.78) ***	1.21 (0.88–1.54) ***	0.66 (0.49–0.87)	0.52 (0.27–0.81)
SP-B	0.27 (0.14–0.41)	0.22 (0.14–0.32)	0,28 (0–0.62)	0,20 (0.05–0.39)	0.13 (0–0.40)
SP-C	0.48 (0.42–0.54)	0.87 (0.75–1.02) ***	1.71 (1.10–2.41) ***	0,77 (0.63–0.91)	1.26 (1.01–1.49) *
SP-D	6.03 (5.52–6.54)	7.16 (6.11–8.30)	9.29 (5.33–14.64)	6.21 (4.57–7.75)	11.51 (5.59–21.16)
CC	3 (2–4)	9 (4–13)	13 (9–17)	2 (1–3)	2 (0–33)
Lac	1.60 (1.53–1.67)	1.55 (1.49–1.61)	2.12 (1.89–2.35)	1.86 (1.78–1.94)	1.48 (1.26–1.70)
Glu	3.55 (3.54–3.56)	3.78 (3.41–4.15)	3.16 (2–86–3.46)	4.25 (4.02–4.48)	6.05 (1.9–10.1)
Total Protein	0.36 (0.34–0.38)	0.11 (0.08–0.13)	0.43 (0.33–0.53)	0.40 (0.31–0.49)	0.22 (0.13–0.31)

In routine CSF examination, only total CSF protein concentration showed a significant reduction in AQS and VIIP patients (p = 0.01) compared to controls. Compared to control, SP-A values were significantly elevated in all types of acute CSF circulation disturbance (AQS and acute HC w/o AQS; p<0.001). SP-A levels in NPH were slightly elevated compared to controls, but the difference did not reach statistical significance (p = 0.069) (Fig 1A). Compared to controls, SP-C levels were elevated in patients with AQS, acute hydrocephalus without AQS (both p<0.001) and pseudotumor cerebri (p<0.001). There was no significant alteration in NPH patients (Fig 1C). Reliably measurable SP-B levels were found 17 out of 126 CSF samples, mostly in the control group (12 samples revealed concentrations in the range between 0.67 ng/ml and 1.915 ng/ml). In 109 CSF samples SP-B was below the detection limit (Fig 1B). SP-D was detected in patients and controls without significant statistical differences, although a clear trend was observable between the group of pseudotumor cerebri and controls (p = 0.056) (Fig 1D).

**Fig 2:**
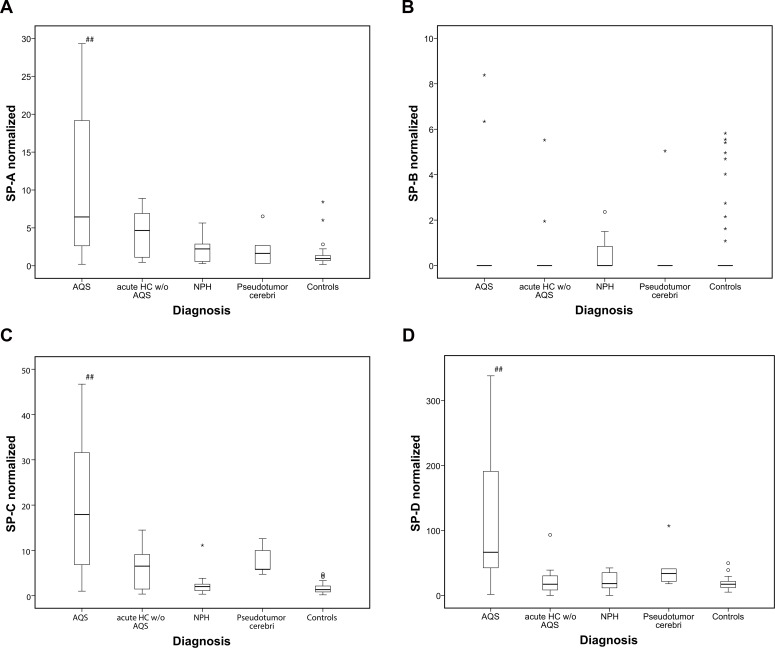
a-d) CSF levels of SP A—D (ng/ml) in control group and hydrocephalus subgroups normalized to total CSF protein concentration of the patients. Significant elevation of SP levels was found for SP-A, C and D for AQS patients compared to control only. Other hydrocephalus entities showed trends towards elevation only.*: Data value lies >3 times of the interquartile range away from the mean value.°: Data value lies between 1.5 and 3x of the interquartile range away from the median value.

**Fig 3:**
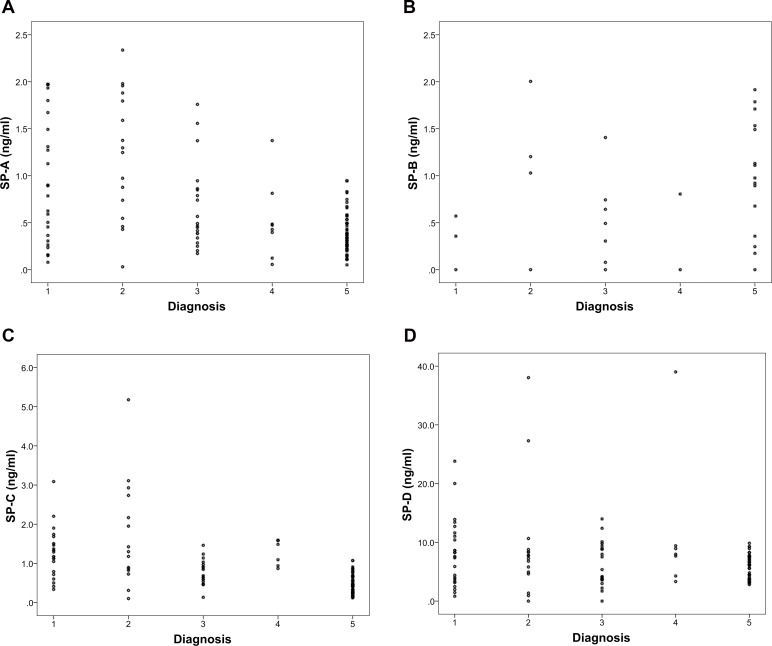
a—d) Dotplot Charts for CSF levels of SPs A-D (ng/ml) in control group and hydrocephalus subgroups normalized to total CSF concentration showing the individual data values for each specimen. Significance levels: # p<0.05 ## p<0.001; all vs. Control. *: Data value lies >3 times of the interquartile range away from the mean value. °: Data value lies between 1.5 and 3x of the interquartile range away from the median value.

Additionally, all SP values were normalized to total CSF protein concentration. However, 29 specimens had to be excluded due to missing total protein concentrations and small sample volume, thus resulting in a reduced total of samples of the different groups (49 controls, 22 AQS, 13 acute HC w/o AQS and 11 NPH specimen). Results of the normalized SPs are shown in Fig 2A–2D and as dotplots in Fig 3A–3D. Subsequently, comparison of values between investigated groups (AQS, acute HC w/o AQS, NPH, Pseudotumor cerebri and controls) by means of ANOVA revealed statistically significant differences for SP-A (p<0.001), SP-C (p<0.001) and SP-D (p<0.001). No differences were found for SP-B (p = 0.986). Post hoc analysis using a two-sided Dunnett test revealed that mean values of SP-A, SP-C and SP-D from AQS patients were statistically different from the control group. No statistically significant differences were found for NPH, acute HC w/o AQS and Pseudotumor cerebri values compared to the control group.

**Fig 4:**
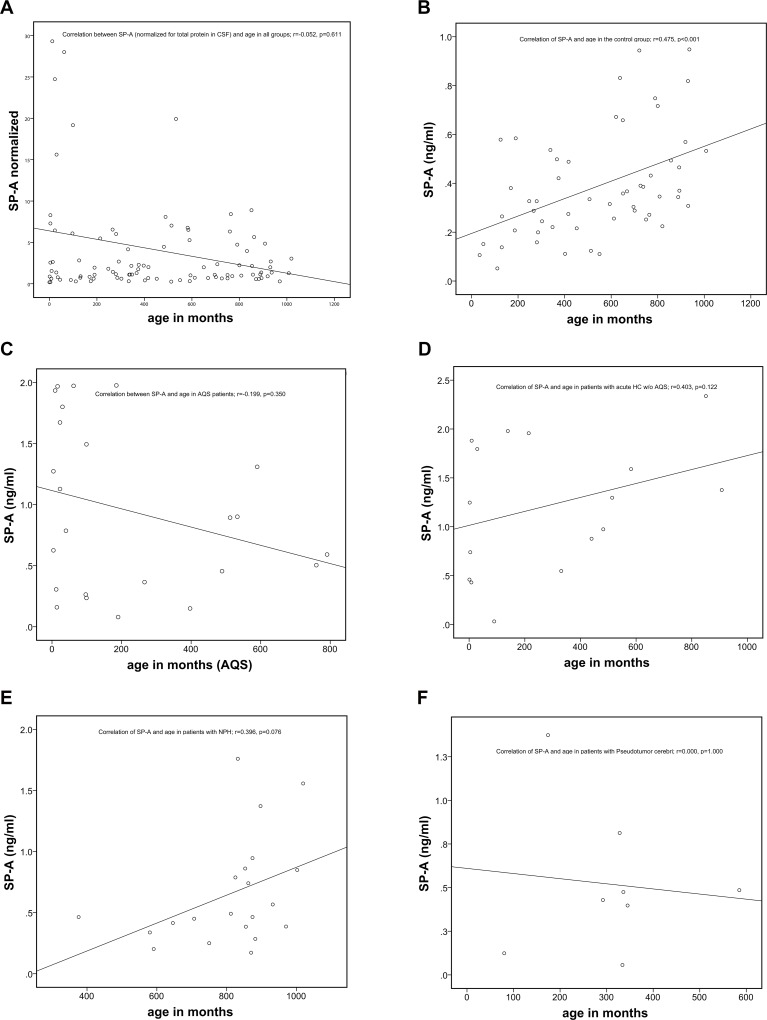
SP-A levels increase with age. a) No correlation of normalized SP-A concentrations of all 126 specimens (r = 0.059, p = 0.519). b) Positive age correlation of SP-A within the control group (r = 0.475, p<0.01). c)–f) Plots of age correlation for individual patient subgroups.

SP-A showed a correlation to the age of the subjects in the control group only (r = 0.478; p < 0.001; Fig 4B). There was no a significant association of SP-A values and age for the entire set of samples of 126 patients (r = 0.059, p = 0.519, Fig 4A). Age did also not correlate with SP-A levels in the subgroups of different pathological entities (Fig 4C–4F). The other SPs did not show any correlation with age.

## Discussion

In this study we quantified the concentration of SP in the CSF of human control patients in comparison to patients suffering from AQS, acute hydrocephalus without AQS, NPH and pseudotumor cerebri. We were able to demonstrate significant changes of SP-A and SP-C in patients suffering from AQS and acute hydrocephalus without AQS. Significant changes of SP-C were also found in patients suffering from pseudotumor cerebri. Furthermore there was a delineable tendency of elevated SP-D in patients with pseudotumor cerebri, but the difference compared to the control group did not reach statistical significance. We also noticed a trend of increased SP-A in patients with NPH although this did not reach significance. Furthermore we could show a significant increase of SP-A in correlation with age in normal subjects.

SP-A, besides its immunological functions, stabilizes the pulmonary surfactant layer and contributes to its surface tension lowering activity [2,18–29]. SP-C strongly interacts with surfactant phospholipids, is responsible for initial surfactant film formation and thus also contributes to the reduction of surface tension [7]. Taken together, both proteins play a pivotal role for the regulation of surface tension by maintaining the rheological properties of the intraalveolar fluid layer.

It is well known that surfactant protein production and secretion depend on the impact of mechanical force on the alveolar epithelium [30]. Deep tidal volumes result in increased SP secretion and consequently reduced intraalveolar surface tension [31–33].

Concerning the CNS, we recently identified the epithelium of the choroid plexus and the ependymal cells as cellular sources of SP-A and SP-C ^10^. Considering these facts we hypothesize that both—SP-A and SP-C—in physiological analogy to their pulmonary functions—participate in the regulation of the CSF rheological properties in the central nervous system. Hydrocephalic conditions like AQS and acute HC without AQS occur due to disturbed CSF flow or restricted CSF resorption and finally lead to increased pressure within the CSF spaces and ventricular enlargement. Since in the lungs the production rate of SP-A and SP-C by the alveolar epithelium increases corresponding to enhanced transepithelilal forces (for example following deep inspiration), it might be that a similar mechanism exists in the brain [30]. Assuming that, it seems possible that disturbed CSF flow and restricted CSF resorption, both being substantial pathomechanistic elements of the aforementioned hydrocephalic conditions, increase mechanical force to the ependyma and the choroid plexus by increasing ICP pulse amplitude. Thus, it may increase SP production possibly downstream to strain on the actin skeleton as postulated by Han and colleagues [30,34].

Interestingly, CSF SP-A levels showed a significant increase in correlation with age–exclusively in the control group. Since SP-A CSF levels are increased in all disease subgroups, it seems likely that the pathophysiological alterations of CSF SP-A obscure the identifed association with age. Furthermore, the small sample size of disease subgroups also contributes in dimishing this phenomenon. Arani and coworkers were able to demonstrate in vivo that the stiffness of the brain increases in older age [35]. Increased stiffness of brain parenchyma the arterial blood flow shockwave will result in an increased ICP amplitude, hypothesized as a main contributor to CSF secretion in the ventricles, resulting in intermittently increased transependymal mechanical forces [11]. The resulting increased strain of ependymal cells in conjunction with ventricular enlargement may cause elevated SP-A secretion as a counter regulatory mechanism. Although statistical significance was not achieved, a trend of increased SP-A in NPH patients was noticeable. Unlike in AQS and acute HC without AQS the underlying pathomechanisms (reduced vascular compliance and decreased CSF resorption through the arachnoidal granulations causing elevated ICP pulse amplitudes and finally a characteristic pattern of CSF space enlargement) of NPH are rather chronic processes than sudden events and resulting disturbances of CSF-flow can therefore be partially compensated over the course of the disease [36]. Possible regulatory adaptions of SP-A secretion might thus be less obvious [11,14,37,38].

In addition to AQS and acute HC without AQS SP-C levels were also elevated in patients suffering from pseudotumor cerebri. Pseudotumor cerebri is characterized by an elevated intracranial pressure but absence of ventricular enlargement [17,39]. A common characteristic feature of AQS, acute HC without AQS and pseudotumor cerebri is elevated intracranial pressure. Since pseudotumor cerebri is the only pathology investigated in this study accompanied by elevated intracranial pressure without ventriculomegaly we hypothesize that SP-C secretion is predominantly increased downstream to increased intracranial pressure.

Interestingly SP-A was only increased in pathologies with ventricular enlargement but did not reveal elevation in pseudotumor cerebri. This might indicate that dilation of the inner CSF spaces is the primary stimulus for SP-A secretion, probably succeeding periods of increased intracranial pressure without ventriculomegaly yet having occurred.

The results of our SP-B measurements in the different groups seem inconsistent. 17 of 126 CSF samples revealed SP-B concentrations within the detection range of the ELISA system, 109 samples revealed concentrations below the guaranteed detection limit. The majority of detectable SP-B levels were found in the control group (12 out of 60 controls). In our previous study we were able to demonstrate production of SP-B in the human CNS and its abundance in CSF with western blot and ELISA. Considering the issue of the detection limit of the used ELISA system and the small sample size, further studies must be stressed to investigate the importance of SP-B for CSF homeostasis.

SP-D was detected in all CSF samples. A trend of increased SP-D was observable in the pseudotumor cerebri group, but the difference did not reach statistical significance compared to our control cohort. Since the number of patients suffering from pseudotumor cerebri in our study was comparatively small (n = 6) but the difference in SP-D CSF levels compared to controls almost achieved statistical significance we hypothesize that SP-D alteration might play a role in pathophysiology of pseudotumor cerebri. To corroborate this hypothesis further studies with a larger cohort of pseudotumor cerebri patients are necessary.

Normalising the SP concentrations to the absolute CSF protein content led mainly to a significant reduction of the sample group for NPH (n = 11), but also for the other entities. The normalization further substantiates that SP concentrations in AQS are highly elevated compared to controls. However, due to the small sample size of other pathological entities, only trends to SP elevation were found in PC patients. The results of the normalization additionally indicate that the production of SPs does not simply follow the (etiology dependent) trend of total CSF protein content. Down regulation of total CSF protein might be a counterregulatory measure to improve reabsorption of CSF while surfactant proteins are maintained or even elevated to support CSF flow and lower surface tension at the brain-CSF interface. Furthermore it demonstrates that surfactant proteins are not present within the CSF due to a passive filtration of serum proteins across the BBB.

The major shortcomings of the study are the small size of the control group and the lack of follow-up surfactant protein levels after therapy (e.g. after shunt placement in NPH patients) to evaluate therapeutic effects on the cerebral surfactant system. Obtaining such data will be an ethical problem that can only be solved with randomised study protocols justified when more data become available.

The identification of altered SP concentration in CSF as pathophysiological mechanism, counterregulatory effect or pure epiphenomenon is difficult. Further investigations require studies focusing e.g. on animal models and on the clinical progress of a particular condition over time (AQS, NPH, acute hydrocephalus, pseudotumor cerebri) in correlation to changing SP concentrations in the CSF.

## Conclusion

The present study demonstrated the presence of Surfactant Proteins A, B, C and D under physiological conditions in human CSF. SP-A and SP-C are elevated in CSF samples of patients suffering from conditions with altered CSF dynamics. Elevated SP-A secretion may predominantly be caused by ventricular enlargement, whereas SP-C might predominantly be elevated due to increased intracranial pressure. These hypotheses require further investigations of underlying pathomechanisms and signaling cascades. Our findings, however, suggest a potential use of SP-A and SP-C as diagnostic markers in diseases with ventriculomegaly and elevated intracranial pressure.
